# Clinical characteristics and prognosis of Talaromycosis marneffei associated immune reconstitution inflammatory syndrome in AIDS patients

**DOI:** 10.1371/journal.pntd.0012609

**Published:** 2024-10-18

**Authors:** Qinzhi Zhang, Huihua Zhang, Pengle Guo, Weiyin Lin, Feilong Xu, Xiaoping Tang, Linghua Li

**Affiliations:** 1 Guangzhou Medical Research Institute of Infectious Diseases, Guangzhou Eighth People’s Hospital, Guangzhou Medical University, Guangzhou, China; 2 Infectious Disease Center, Guangzhou Eighth People’s Hospital, Guangzhou Medical University, Guangzhou, China; 3 Institute of Infectious Diseases, Guangzhou Eighth People’s Hospital, Guangzhou Medical University, Guangzhou, China; Xuzhou Medical University, CHINA

## Abstract

**Background:**

Immune reconstitution inflammatory syndrome (IRIS) is an inflammatory reaction that occurs in HIV/AIDS patients after antiretroviral therapy (ART) initiation. Along with immune system recovery, IRIS can overreact to existing infections or latent pathogens, causing symptoms that mimic those infections. Few studies elucidated the clinical features and prognosis of Talaromycosis marneffei (TSM)-associated IRIS in HIV/AIDS patients. The aim of our study was to evaluate the incidence, clinical characteristics, and prognosis of TSM-associated IRIS by retrospectively analyzing the clinical data of HIV/AIDS patients with TSM.

**Methodology/Principal findings:**

A total of 224 HIV/AIDS inpatients with TSM were enrolled, aged between 19 and 81 years. Among them, 86.6% were male and 13.4% were female, of which 24 (10.7%) patients developed IRIS. In IRIS group, the median time from ART initiation to IRIS occurrence was 9.0 days (IQR, 5.0–16.8 days), with 87.5% (21/24) occurring within 2 weeks. Primary clinical manifestations included recurrent fever and exacerbation of pulmonary infection. At the onset of IRIS, 54.2% (13/24) patients were treated with intravenous dexamethasone, and 12.5% (5/24) patients were treated with oral prednisone for 1–3 weeks. No significant differences in baseline characteristics or ART regimens were observed between IRIS and non-IRIS groups; however, patients in IRIS group had higher levels of CRP, CD4^+^ count, and CD4^+^/CD8^+^ ratio than non-IRIS group (equivalent time point: 1–2 weeks after ART initiation) at IRIS onset. The IRIS group exhibited longer hospital stays and higher readmission rates, but equivalent mortality rates compared with non-IRIS group.

**Conclusions/Significance:**

IRIS is a common complication in HIV/AIDS patients with TSM, often occurring within 2 weeks after ART initiation and exhibiting more pronounced immune reconstitution. The occurrence of IRIS significantly extended the hospitalization duration and increased the rate of readmission but had no influence on the mortality rate.

## Author summary

Immune reconstitution inflammatory syndrome (IRIS) is an inflammatory reaction that occurs in HIV/AIDS patients after antiretroviral therapy (ART) initiation. Along with immune system recovery, IRIS can overreact to existing infections or latent pathogens, causing symptoms that mimic those infections. The clinical characteristics of IRIS depend on the causative pathogen. Talaromycosis marneffei (TSM) is one of the common opportunistic infections in HIV/AIDS patients, and often presents a disseminated form of infection, affecting multiple organs throughout the body. However, few reports on TSM-associated IRIS lead to challenges in clinical management. In this study, we carried out a retrospective analysis of TSM-associated IRIS, elucidated the occurrence and clinical features of TSM-associated IRIS, and compared the laboratory examination data and outcomes in TSM patients with or without IRIS. This study aims to review clinical data from HIV/AIDS patients with TSM to assess the frequency, features, and outcomes of TSM-associated IRIS, to improve understanding of the condition, and to facilitate clinical treatment effectively.

## Introduction

Talaromycosis marneffei (TSM) is one of the most common opportunistic infections in HIV/AIDS patients in Southeast Asia and southern China [1]. In highly endemic areas, the prevalence of *Talaromyces marneffei (T*. *marneffei)* infection among HIV/AIDS inpatients accounts for 16.1%, thereby contributing to a high mortality rate [2]. HIV/AIDS patients complicated by TSM often present a disseminated form of infection, affecting multiple organs throughout the body. Common clinical manifestations include fever, skin lesions, and hepatosplenomegaly [3].

Immune reconstitution inflammatory syndrome (IRIS) refers to a paradoxical inflammatory response that occurs following the initiation of antiretroviral therapy (ART) in patients with immunodeficiency, particularly those with HIV/AIDS. As the immune system begins to recover and regain functionality, an exaggerated inflammatory reaction can occur against previously acquired opportunistic infections or latent pathogens. This syndrome often manifests with clinical symptoms similar to those of the underlying infections, leading to a complex interplay between immune recovery and inflammation. Understanding IRIS is crucial for optimizing treatment strategies and managing patient outcomes effectively [4]. Different types of infection-associated IRIS exhibit distinct incidence rates, clinical features, and prognoses. Reported rates of IRIS associated with invasive fungal diseases were 10–45% for cryptococcal meningitis, 4–12.4% for pneumocystis pneumonia, and 2.4–11.3% for candidiasis [5–9]. Cryptococcal meningitis-associated IRIS often presents severe headaches, raised intracranial pressure, together with impaired clinical nerve function, and greatly increases mortality [10]. Pneumocystis pneumonia-associated IRIS presents the reappearance of fever and dyspnea, causes respiratory failure in severe cases, but does not lead to high mortality [11]. The clinical characteristics and prognosis of TSM-associated IRIS remain unclear. So far, only 9 cases of TSM-associated IRIS have been reported, involved in Vietnam, Thailand, India, the United Kingdom, and China. All these cases had a history of residence or travel in TSM epidemic regions [12–18]. The limited knowledge leads to misdiagnosis and potential confusion regarding treatment failure or adverse drug reactions. This study aims to evaluate the occurrence, clinical features, and prognosis of TSM-associated IRIS by retrospectively analyzing the clinical data of HIV/AIDS patients with TSM, in order to enlarge the understanding of TSM-associated IRIS and improve TSM-associated IRIS diagnosis and treatment.

## Material and methods

### Ethics statement

This study was approved by the Ethical Committee of the Guangzhou Eighth People’s Hospital, Guangzhou Medical University (Approval No.202332269). Informed consent was provided by all participants.

### Patients

A retrospective study was conducted among HIV/AIDS patients with TSM from June 2021 to October 2023 who initiated or restarted ART at Guangzhou Eighth People’s Hospital (Guangdong Province, China). This study was a cross-sectional survey to investigate the incidence of TSM-associated IRIS. According to other fungal disease-associated IRIS studies, the incidence of TSM-associated IRIS was estimated to 8%. A tolerable error of 4% and a confidence level of 1- α = 0.95 were specified, and a minimum sample size of N = 202 cases to be investigated was calculated using PASS 11 software. The inclusion criteria were: (1) diagnosis of HIV-1 infection; (2) diagnosis of TSM: culture positive of *T*. *marneffei* either in blood or bone marrow, or histopathological positive in lymph nodes; (3) naïve to ART or restarted ART (stopped ART >3 months). The exclusion criteria were: (1) follow-up time less than one week after ART initiation; (2) patients with malignant tumors or pregnant.

### Case definitions

TSM-associated IRIS was diagnosed as follows: (1) the exacerbation of clinical symptoms specifically attributed to TSM after ART initiation, and (2) exclusion of the possibility of treatment failure, drug-related adverse effects, and new infections. The diagnosis of TSM-associated IRIS requires both (1) and (2) to be met, and all diagnoses are made independently by two experienced physicians. IRIS events were defined within 3 months of ART. Exacerbation of pulmonary infections is defined as a worsening of imaging changes, including increased pleural effusion, increased density of lung shadows, and increased extent of lesions on the current CT compared to the previous one. Increased lymphadenopathy is defined as a new lymph node enlargement or an increase in the number or enlargement of existing lymph nodes.

### Data collection and analysis

Data were obtained from the hospital’s electronic medical record system and manually calibrated and reviewed. Medical history collection, laboratory data testing, and etiological testing were carried out by specialists. The preferred antifungal treatment regimen consists of amphotericin B for induction therapy, followed by itraconazole for consolidation. For patients intolerant to amphotericin B, voriconazole should be used as an alternative, with careful monitoring for adverse drug reactions. ART should be initiated subsequently, with ongoing evaluation of treatment efficacy and monitoring for complications such as IRIS. The time of admission was taken as baseline, and clinical information of HIV/AIDS patients with TSM was retrospectively collected, including (1) demographic characteristics: sex and age; (2) concurrent opportunistic infections: *Mycobacterium tuberculosis*, non-tuberculous mycobacteria, *Pneumocystis jirovecii*, cryptococcus, cytomegalovirus, and candida; (3) laboratory parameters: CD4^+^ count, CD4^+^/CD8^+^ ratio, HIV RNA load, C-reactive protein (CRP), white blood cells (WBC), monocytes (MONO), hemoglobin (HGB), platelets (PLT), procalcitonin (PCT), albumin (ALB), aspartate aminotransferase (AST), alanine aminotransferase (ALT); and (4) treatment and prognosis: ART initiation date, ART regimen, duration of hospitalization, mortality rate, and readmission rate. The endpoint was the occurrence of IRIS, death, or loss of follow-up.

### Statistical analyses

Patients were classified into IRIS and non-IRIS groups depending on whether IRIS occurred or not after ART initiation. The time point of 1–2 weeks after ART initiation in the non-IRIS group was considered the equivalent time point for IRIS occurrence. Continuous features were expressed as median with interquartile range (IQR) or mean with standard deviation (SD), and for categorical variables as frequency (percentage). Chi-square test or Mann–Whitney U test was used to compare categorical and continuous variables between two groups, respectively. Data across three points were analyzed using Kruskal-Wallis test in IRIS group. Statistical significance was considered as *P*-value <0.05. All statistical analyses were performed using IBM SPSS Statistics for Windows 25.0 software.

## Results

### Patient enrollment

**Fig 1:**
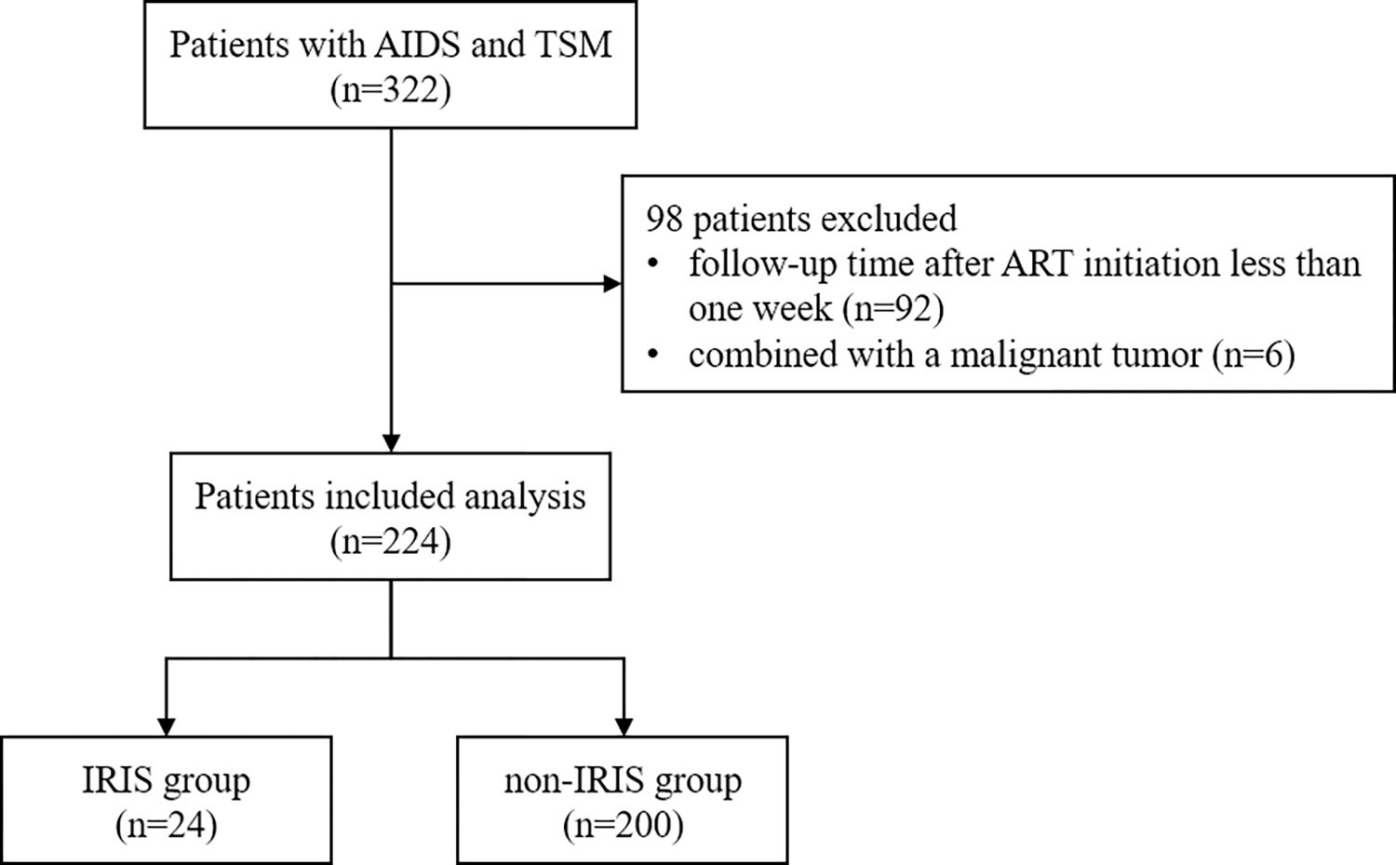
Study design and participants included in the study. TSM: Talaromycosis marneffei, IRIS: immune reconstitution inflammatory syndrome.

A total of 322 HIV/AIDS patients with TSM were enrolled. In these cases, 92 were excluded due to less than one week of follow-up after ART initiation and 6 were excluded due to combined malignancies. The remaining 224 patients were included in the analysis, ranging from 19 to 81 years old, 86.6% were male and 13.4% were female. Among them, 24 (10.7%) patients who developed IRIS were defined as IRIS group, and 200 patients without IRIS were defined as Non-IRIS group (Fig 1).

### Clinical and laboratory characteristics of the IRIS group

**Fig 2:**
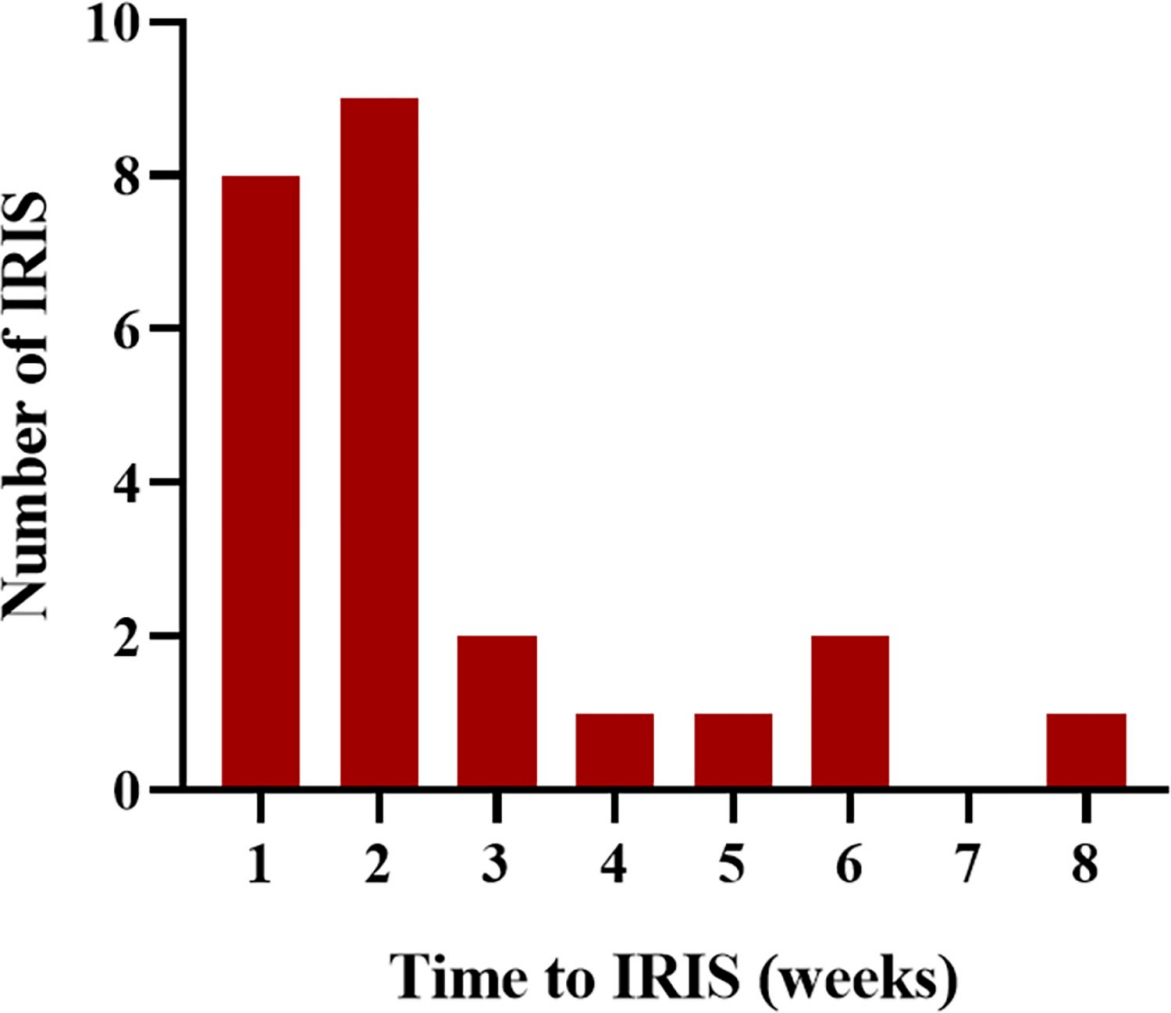
Histogram showing the number of patients who developed IRIS over time after ART initiation.

**Fig 3:**
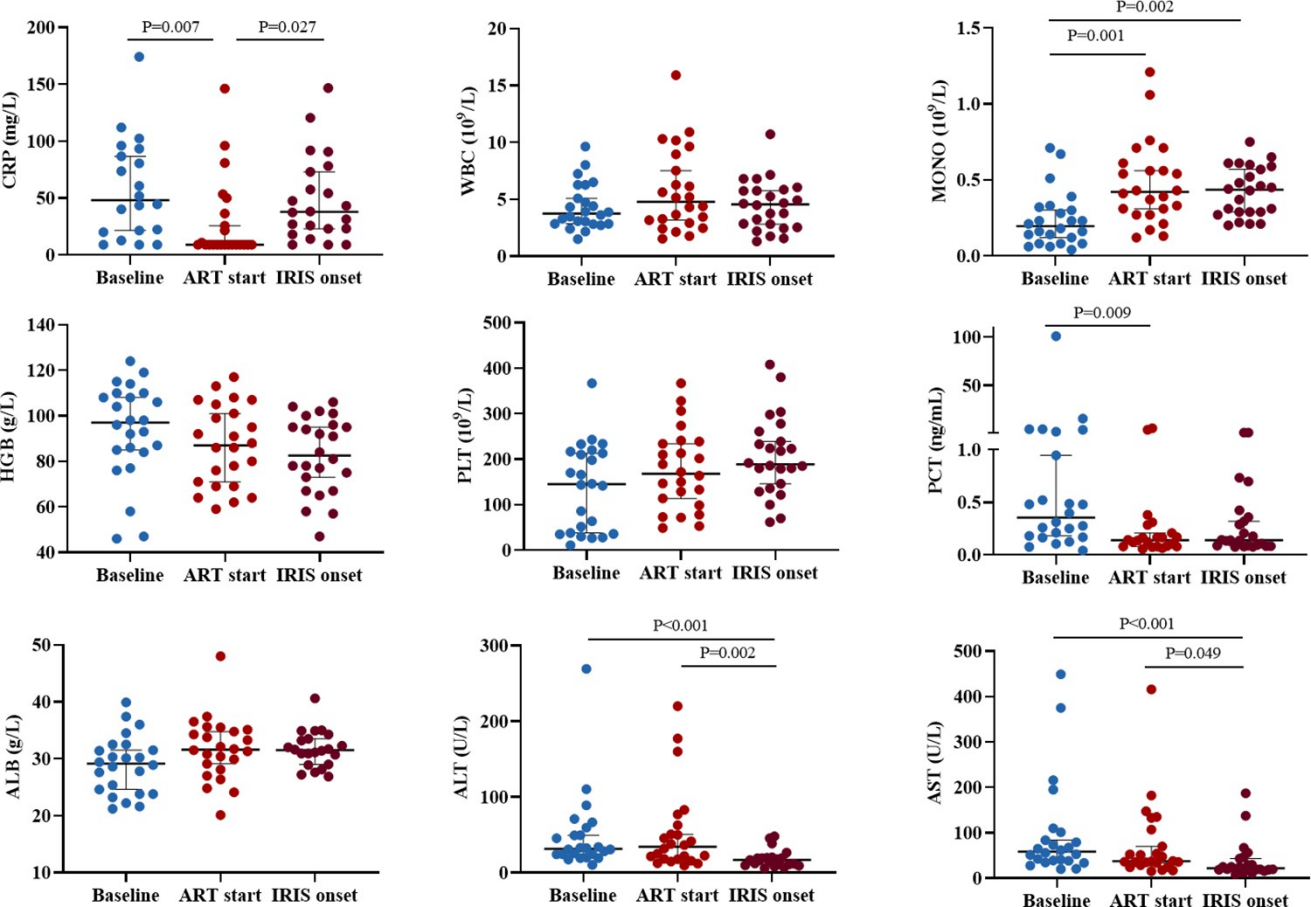
Comparisons of laboratory variables at baseline, ART initiation, and IRIS onset in IRIS group. Significant differences (P < 0.05) between different time points are indicated. ART; antiretroviral therapy; IRIS, immune reconstitution inflammatory syndrome. CRP: C-reactive protein, WBC: white blood cell, MONO: monocytes, HGB: hemoglobin, PLT: platelets, PCT: procalcitonin, ALB: albumin, ALT: alanine aminotransferase, AST: aspartate aminotransferase.

In 24 IRIS patients, the median occurrence time was 9.0 days (IQR, 5.0–16.8 days) after ART initiation. Among them, 70.9% (21/24) occurred within 2 weeks of ART initiation (Fig 2). Primary clinical manifestations of IRIS included recurrent fever and exacerbation of pulmonary infections (Table 1). At IRIS onset, 54.2% (13/24) patients received intravenous dexamethasone for 1 to 3 weeks, 12.5% (5/24) with oral prednisone for 1 to 3 weeks, and 58.3% (14/24) with nonsteroidal anti-inflammatory drugs (S1 Table). All patients were improved and discharged after treatment, with no severe organ failure or death. At IRIS onset, blood culture results of *T*. *marneffei* were 19 negative (79.2%), 2 positive (8.3%), and the other three did not receive blood culture examination (12.5%). We analyzed serial laboratory test data in IRIS group at baseline, ART initiation, and IRIS onset. Compared with baseline, CRP levels decreased significantly at ART initiation (48.3 mg/L vs. 9.0 mg/L, P = 0.007) and then significantly increased at IRIS onset (9.0 mg/L vs. 37.9 mg/L, P = 0.027). Monocyte counts were significantly increased (0.4 ×10^9^/L vs. 0.2 ×10^9^/L, P = 0.001) at ART initiation compared to baseline, and PCT levels were significantly decreased (0.1 ng/ml vs. 0.4 ng/ml, P = 0.009). Decreased ALT and AST levels were expected at IRIS onset compared to ART initiation (16.9 U/L vs. 34.4 U/L, P = 0.002; and 22.5 U/L vs. 37.9 U/L, P = 0.049, respectively). No statistical differences over time for other variables, including WBC, HGB, PLT, and ALB (Fig 3).

**Table 1:** Clinical manifestations related to TSM-associated IRIS in IRIS group.

Signs and symptoms	Patients (n = 24)
**Fever (%)**	18(75.0)
**Exacerbation of pulmonary infection (%)**	16(66.7)
**Abdominal pain (%)**	5(20.8)
**Cough (%)**	4(16.7)
**Lymph node enlargement (%)**	3(12.5)
**Skin lesions (%)**	2(8.3)

The clinical presentations all manifested as an exacerbation or new onset compared to before the occurrence of IRIS. The exacerbation of pulmonary infection has been confirmed through CT scanning.

### Comparison of baseline demographic and clinical characteristics between the IRIS and non-IRIS groups

The IRIS group included 91.7% (22/24) males and 8.3% (2/24) females, with a median age of 36.0 years (IQR, 31.8–46.0 years). The non-IRIS group consisted of 86.0% (172/200) males and 14.0% (28/200) females, with a median age of 37.5 years (IQR, 30.0–48.3 years). In IRIS group, 3 patients (12.5%) were co-infected with *Mycobacterium tuberculosis*, 3 (12.5%) with non-tuberculous mycobacteria, 5 (20.8%) with cytomegalovirus, 9 (37.5%) with *Pneumocystis jirovecii*, and 8 (33.3%) with candida. In non-IRIS group, 12 patients (6.0%) were co-infected with *Mycobacterium tuberculosis*, 9 (4.5%) with non-tuberculous mycobacteria, 52 (26.0%) with cytomegalovirus, 10 (5.0%) with *Pneumocystis jirovecii*, and 43 (21.5%) with candida. No significant differences were observed between two groups regarding demographic characteristics, co-infection status, the interval between antifungal and ART initiation, CD4^+^ counts, and blood test results at baseline (Tables 2 and S2).

**Table 2:** Baseline demographic and clinical characteristics of IRIS group and non-IRIS group.

Characteristic	Total (n = 224)	IRIS (n = 24)	non-IRIS (n = 200)	*P*-value
**Male (%)**	194(86.6)	22(91.7)	172(86.0)	0.651
**Age (years, IQR)**	37.0(30.0,49.3)	36.0(31.8,46.0)	37.5(30.0,48.3)	0.894
**Interval between antifungal and ART initiation (days, IQR)**	12.0(8.0,17.0)	11.5(7.8,16.3)	12.0(8.8,17.0)	0.478
**OIs at the time of ART initiation (%)**				
*Mycobacterium tuberculosis*	15(6.7)	3(12.5)	12(6)	0.402
non-tuberculous mycobacteria	12(5.36)	3(12.5)	9(4.5)	0.218
Cryptococcus	10(4.5)	0(0.0)	10(5.0)	0.572
Cytomegalovirus	57(25.5)	5(20.8)	52(26.0)	0.848
*Pneumocystis jirovecii*	53(23.7)	9(37.5)	44(22.0)	0.068
Candida	51(22.8)	8(33.3)	43(21.5)	0.191
**CD4**^**+**^ **count (cells/μL, IQR)**	13.0(6.0,27.0)	13.0(7.0,46.5)	13.0(6.0,25.25)	0.439
**CD4**^**+**^**/CD8**^**+**^ **ratio (IQR)**	0.06(0.03,0.1)	0.05(0.03,0.09)	0.06(0.03,0.1)	0.843
**HIV RNA (log**_**10**_ **cp/ml, IQR)**	5.6(5.1,6.1)	5.8(5.3,6.1)	5.6(5.1,6.1)	0.493
**CRP (mg/L, IQR)**	51.9(24.9,91.9)	48.3(21.2,88.4)	51.9(25.7,91.9)	0.593
**WBC (10** ^**9**^ **/L, IQR)**	4.0(2.9,5.7)	3.8(2.9,5.4)	4.0(3.0,5.7)	0.693
**MONO (10** ^**9**^ **/L, IQR)**	0.2(0.1,0.3)	0.2(0.1,0.3)	0.2(0.1,0.3)	0.968
**HGB (g/L, IQR)**	89.0(75.0,107.0)	97.0(84.8,108.5)	88.5(74.8,106.3)	0.335
**PLT (10** ^**9**^ **/L, IQR)**	138.5(64.0,204.0)	145.0(37.5,214.0)	135.5(66.5,201.0)	0.743
**PCT (ng/ml, IQR)**	0.5(0.2,2.3)	0.4(0.2,1.3)	0.6(0.2,2.4)	0.366
**ALB (g/L, SD)**	28.1±5.6	28.9±5.1	28.0±5.7	0.426
**ALT (U/L, IQR)**	28.3(17.5,52.5)	31.7(24.5,52.1)	28.1(16.2,52.5)	0.224
**AST (U/L, IQR)**	60.3(31.1,116.7)	58.9(39.3,88.5)	60.3(30.7,123.5)	0.914

IQR: interquartile range, SD: standard deviation, ART: antiretroviral therapy, OI: opportunistic infection, CRP: C-reactive protein, WBC: white blood cell, MONO: monocytes, HGB: hemoglobin, PLT: platelets, PCT: procalcitonin, ALB: albumin, ALT: alanine aminotransferase, AST: aspartate aminotransferase.

### Comparison of ART regimens and laboratory test results between IRIS and non-IRIS groups at ART initiation

Nucleoside reverse transcriptase inhibitors (NRTIs) combined with integrase strand transfer inhibitors (INSTIs) was the widely used ART regimen in both groups, including 62.5% (15/24) in IRIS group and 59.5% (119/200) in non-IRIS group, respectively. We compared laboratory test results at ART initiation and found no significant differences between two groups (S3 Table).

### Comparison of laboratory results between IRIS and non-IRIS groups at IRIS onset

**Fig 4:**
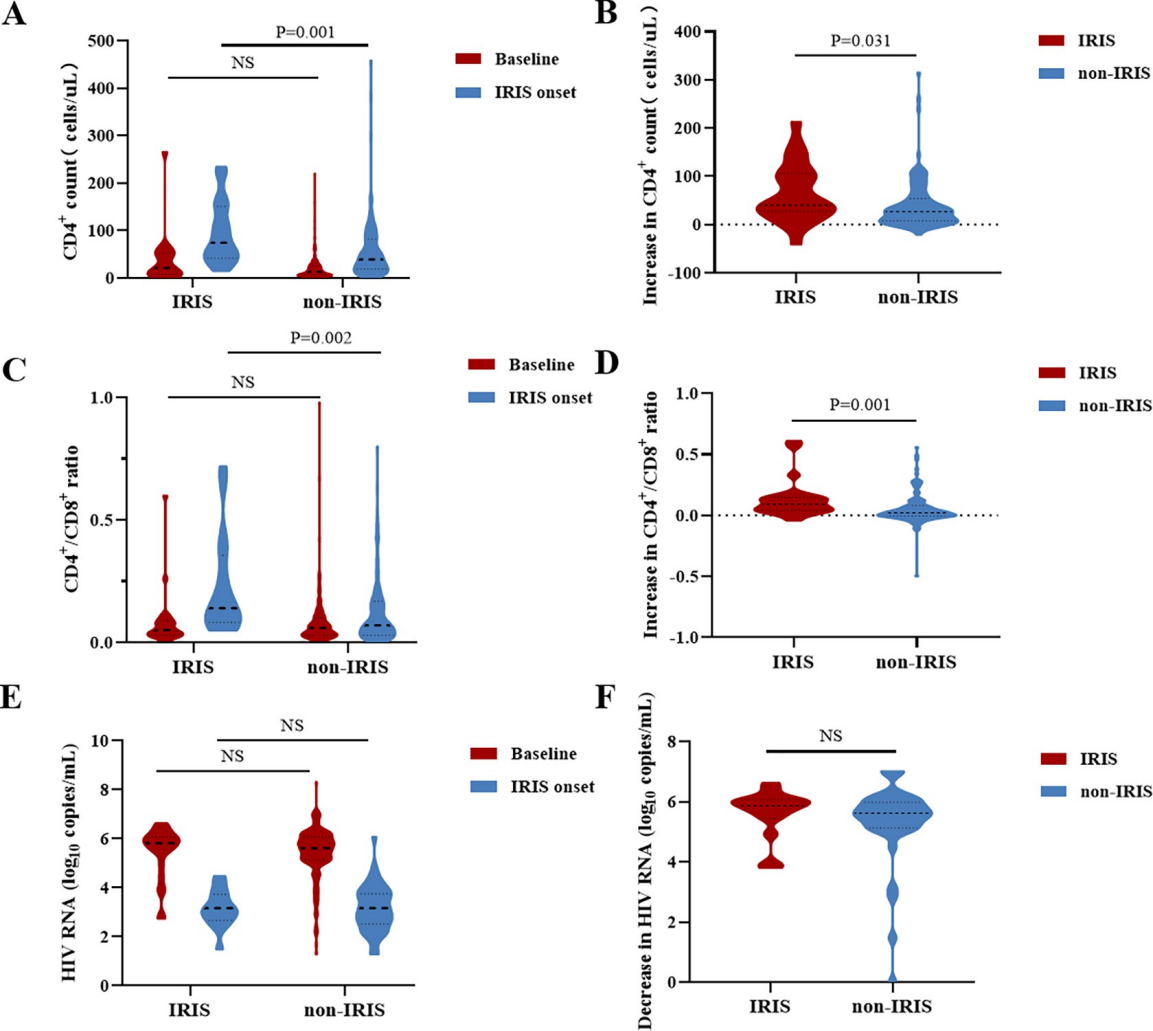
The comparison of CD4^+^ count, CD4/CD8 ratio, and HIV RNA load levels. (A). CD4^+^ count at baseline and IRIS onset. (B). Increase in CD4^+^ count from baseline to IRIS onset. (C). CD4^+^/CD8^+^ ratio at baseline and IRIS onset. (D). Increase in CD4^+^/CD8^+^ ratio from baseline to IRIS onset. (E). HIV RNA load at baseline and IRIS onset. (F). Decrease in HIV RNA load from baseline to IRIS onset. ART: antiretroviral therapy, IRIS: immune reconstitution inflammatory syndrome.

At IRIS onset, CRP levels were higher in IRIS group than non-IRIS group (37.9 mg/L vs. 10.6 mg/L, P = 0.003) (Table 3). The IRIS group also exhibited higher CD4^+^ count (74.5 cells/μL vs. 40.0 cells/μL, P = 0.001) and CD4^+^/CD8^+^ ratio (0.14 vs. 0.07, P = 0.002) at IRIS onset, with no difference in HIV RNA load (Fig 4A, 4C and 4E). When comparing the changes from ART initiation to IRIS onset, IRIS group showed greater increases in CD4^+^ count (40.0 cells/μL vs. 27.0 cells/μL, P = 0.031) and CD4^+^/CD8^+^ ratio (0.09 vs 0.02, P = 0.001) compared to non-IRIS group (Fig 4B, 4D and 4F).

**Table 3:** Comparison of laboratory results between IRIS and non-IRIS groups at IRIS onset.

Characteristic	Total (n = 224)	IRIS (n = 24)	non-IRIS (n = 200)	*P-*value
**CRP (mg/L, IQR)**	11.9(9.0,38.3)	37.9(23.1,69.3)	10.6(9.0,31.1)	0.003^a^
**WBC (10** ^**9**^ **/L, IQR)**	3.8(2.7,5.3)	4.6(2.8,5.8)	3.7(2.8,5.2)	0.462
**MONO (10** ^**9**^ **/L, IQR)**	0.4(0.3,0.5)	0.4(0.3,0.6)	0.4(0.3,0.5)	0.282
**HGB (g/L, IQR)**	81.0(67.0,97.0)	82.5(71.5,95.3)	80.0(67.0,97.0)	0.797
**PLT (10** ^**9**^ **/L, IQR)**	184.0(129.0,253.0)	189.0(143.5,244.5)	183.0(128.5,252.0)	0.513
**PCT (ng/ml, IQR)**	0.1(0.1,0.3)	0.1(0.1,0.3)	0.1(0.1,0.3)	0.230
**ALB (g/L, SD)**	32.2±4.8	31.7±3.2	32.3±5.0	0.687
**ALT (U/L, IQR)**	18.4(11.2,35.2)	16.9(10.9,19.8)	18.6(11.7,35.8)	0.238
**AST (U/L, IQR)**	26.8(19.6,38.7)	22.5(19.2,40.2)	27.3(20.0,38.7)	0.505

IQR: interquartile range, SD: standard deviation, CRP: C-reactive protein, WBC: white blood cell, MONO: monocytes, HGB: hemoglobin, PLT: platelets, PCT: procalcitonin, ALB: albumin, ALT: alanine aminotransferase, AST: aspartate aminotransferase.

a Mann-Whitney U test.

### Comparison of the treatment outcomes between IRIS and non-IRIS groups

Compared with non-IRIS group, IRIS group had a longer hospital stay (41.0 days vs. 33.0 days, P = 0.001). In IRIS group, 12 patients (50.0%) experienced readmission events, compared with 41 patients (20.5%) in non-IRIS group, indicating a significantly higher readmission rate in IRIS group (P = 0.001). No difference in mortality rates between two groups (0 vs. 6.0%, P > 0.05) (Table 4).

**Table 4:** Comparison of the hospitalization duration, readmission rate, and mortality rate between IRIS and non-IRIS groups.

Characteristic	Total (n = 224)	IRIS (n = 24)	non-IRIS (n = 200)	*P-*value
**Hospital stays (days, IQR)**	34.0(27.0,44.3)	41.0(34.8,51.3)	33.0(26.8,43.0)	0.001^a^
**Readmission (%)**	53(23.7)	12(50.0)	41(20.5)	0.001^b^
**Death (%)**	6(2.7)	0	6(3.0)	1.000

a Mann-Whitney U test

b Pearson test.

## Discussion

In this study, we observed more TSM-associated IRIS than previously reported nine cases, suggesting that IRIS is a common complication after ART initiation in HIV/AIDS patients with TSM. We found that TSM-associated IRIS occurred predominantly within 2 weeks after ART initiation, indicating an earlier onset than other IRIS, underscoring the need for vigilant against TSM-associated IRIS soon after ART initiation.

IRIS lacks relatively specific clinical features. A previous study reported 3 cases of TSM-associated IRIS in Vietnam presenting new-onset skin lesions or exacerbation of skin lesions [12]. However, in our study, fever and exacerbation of pulmonary infection were the most common clinical manifestations of TSM-associated IRIS, whereas only two patients experienced exacerbation of skin lesions. Diagnosis of IRIS is difficult due to the lack of characteristic manifestations and diagnostic markers. In the case of suspected IRIS, clinicians must make a comprehensive judgment to exclude the possibility of new infections, treatment failure, and adverse drug reactions.

Previous studies profound immunosuppression in HIV/AIDS patients before ART initiation associated with a higher risk of IRIS [6,7,19–22]. In our study, the majority of patients were severe immunodeficiency. However, no significance was observed in baseline CD4^+^ count between IRIS and Non-IRIS groups. It was reported that INSTIs may increase the risk of IRIS, but we found no differences in ART regimens between two groups [22]. In addition, we analyzed the baseline clinical data of patients with and without TSM-associated IRIS, and no variables were significantly associated with the risk of IRIS.

Cryptococcal meningitis-associated IRIS can lead to adverse events in the central nervous system and increase mortality [20, 23]. In the study, IRIS patients showed improvement after active treatment and a similar mortality rate compared with patients without IRIS. However, the occurrence of TSM-associated IRIS led to longer hospital stays, higher readmission rates, and more complicated disease progression. Our study elucidated a clearer understanding of TSM-associated IRIS, facilitating early clinical identification and intervention, and decreasing unnecessary tests and treatment, thus improving effective clinical management.

This study had some limitations. First, patients in our study were enrolled in a single hospital, leading to a selection bias. Second, limited by the lack of standardized diagnostic criteria for TSM-associated IRIS, we were unable to enroll a large sample size of this population in our study. For patients with more co-infections other than TSM, subgroup analyses were difficult to perform due to limited population. More patients are needed for subgroup analysis and risk factors associated with IRIS.

In conclusion, our study found that a considerable proportion of HIV/AIDS patients with TSM experienced IRIS after ART initiation, predominantly in the early stages, and manifested worse nonspecific clinical features. The occurrence of TSM-associated IRIS prolonged hospitalization, increased readmission rates, and exacerbated patients’ economic and disease burdens. Further research on TSM-associated IRIS is urgently needed for its early diagnosis and effective prevention.

## Supporting information

S1 Table Treatment of TSM-associated IRIS. (DOCX)

S2 Table Results of logistic analysis at baseline in the IRIS and non-IRIS groups. (DOCX)

S3 Table Comparison of ART regimen and laboratory test results between IRIS group and non-IRIS group at ART initiation. (DOCX)

S1 Data Excel spreadsheet containing, in separate sheets, the underlying numerical data and statistical analysis for Figs 2, 3, and 4, and Tables 2, 3, 4 and S3. (XLSX)
