# Supplementary material for: Clinical characteristics and prognosis of Talaromycosis marneffei associated immune reconstitution inflammatory syndrome in AIDS patients
Source: PLoS Negl Trop Dis. 2024 Oct 18;18(10):e0012609. doi: 10.1371/journal.pntd.0012609 (PMC11524464; doi:10.1371/journal.pntd.0012609)
Supplement: S1 Table — (DOCX) [file pntd.0012609.s001.docx]

**S1 Table Treatment of TSM-associated IRIS**

| **Treatment for IRIS** | **Patients (n=24)** |
| --- | --- |
| **Intravenous dexamethasone, for 1-3 weeks (%)** | 13(54.2) |
| **Oral prednisone, for 1-3 weeks (%)** | 5(12.5) |
| **Nonsteroidal anti-inflammatory drugs (%)** | 14(58.3) |
| **Other symptomatic supportive treatment (%)** | 6(25.0) |
