# Supplementary material for: Clinical characteristics and prognosis of Talaromycosis marneffei associated immune reconstitution inflammatory syndrome in AIDS patients
Source: PLoS Negl Trop Dis. 2024 Oct 18;18(10):e0012609. doi: 10.1371/journal.pntd.0012609 (PMC11524464; doi:10.1371/journal.pntd.0012609)
Supplement: S2 Table — (DOCX) [file pntd.0012609.s002.docx]

**S2 Table Results of logistic analysis at baseline in the IRIS and non-IRIS groups**

| **Characteristic** | **IRIS (n=24)** | **non-IRIS (n=200)** | **OR (95%CI)** | ***P*-value** |
| --- | --- | --- | --- | --- |
| **Age (years, IQR)** | 36.0(31.8,46.0) | 37.5(30.0,48.3) | 1.002 (0-961-1.044) | 0.936 |
| **Interval between antifungal and ART initiation (days, IQR)** | 11.5(7.8,16.3) | 12.0(8.8,17.0) | 0.994 (0.926-1.067) | 0.873 |
| **CD4^+^ count (cells/μL, IQR)** | 13.0(7.0,46.5) | 13.0(6.0,25.25) | 1.013 (0.997-1.028) | 0.103 |
| **CD4^+^/CD8^+^ ratio (IQR)** | 0.05(0.03,0.09) | 0.06(0.03,0.1) | 0.279 (0.001-58.982) | 0.640 |
| **HIV RNA (log_10_ cp/ml, IQR)** | 5.8(5.3,6.1) | 5.6(5.1,6.1) | 1.072 (0.652-1.761) | 0.784 |
| **CRP (mg/L, IQR)** | 48.3(21.2,88.4) | 51.9(25.7,91.9) | 1.002 (0.989-1.015) | 0.814 |
| **WBC (10^9^/L, IQR)** | 3.8(2.9,5.4) | 4.0(3.0,5.7) | 0.990 (0.746-1.315) | 0.946 |
| **MONO (10^9^/L, IQR)** | 0.2(0.1,0.3) | 0.2(0.1,0.3) | 0.489 (0.007-32.669) | 0.738 |
| **HGB (g/L, IQR)** | 97.0(84.8,108.5) | 88.5(74.8,106.3) | 1.000 (0.973-1.028) | 0.983 |
| **PLT (10^9^/L, IQR)** | 145.0(37.5,214.0) | 135.5(66.5,201.0) | 0.998 (0.992-1.005) | 0.640 |
| **PCT (ng/ml, IQR)** | 0.4(0.2,1.3) | 0.6(0.2,2.4) | 0.952 (0.829-1.093) | 0.486 |
| **ALB (g/L, SD)** | 28.9±5.1 | 28.0±5.7 | 1.050 (0.926-1.190) | 0.446 |
| **ALT (U/L, IQR)** | 31.7(24.5,52.1) | 28.1(16.2,52.5) | 0.998 (0.980-1.017) | 0.839 |
| **AST (U/L, IQR)** | 58.9(39.3,88.5) | 60.3(30.7,123.5) | 1.001 (0.992-1.010) | 0.830 |
