# Supplementary material for: Clinical characteristics and prognosis of Talaromycosis marneffei associated immune reconstitution inflammatory syndrome in AIDS patients
Source: PLoS Negl Trop Dis. 2024 Oct 18;18(10):e0012609. doi: 10.1371/journal.pntd.0012609 (PMC11524464; doi:10.1371/journal.pntd.0012609)
Supplement: S3 Table — (DOCX) [file pntd.0012609.s003.docx]

**S3 Table Comparison of ART regimen and laboratory test results between IRIS group and non-IRIS group at ART initiation.**

| **Characteristic** | **Total (n=224)** | **IRIS (n=24)** | **non-IRIS (n=200)** | ***P-*value** |
| --- | --- | --- | --- | --- |
| **ART regimen (%)** |  |  |  |  |
| NRTIs+ INSTIs | 134(59.8) | 15(62.5) | 119(59.5) | 0.413 |
| NRTIs+ INSTIs+ Albuvirtide | 80(35.7) | 7(29.2) | 73(36.5) |  |
| NRTIs+ NNRTIs | 8(3.6) | 2(8.3) | 6(3.0) |  |
| other | 2(0.9) | 0(0.0) | 2(1.0) |  |
| **CRP (mg/L, IQR)** | 9.0(9.0,29.3) | 9.0(9.0,31.1) | 9.0(9.0,28.7) | 0.782 |
| **WBC (10^9^/L, IQR)** | 3.9(2.6,5.6) | 4.8(3.1,7.9) | 3.9(2.5,5.2) | 0.076 |
| **MONO (10^9^/L, IQR)** | 0.3(0.2,0.5) | 0.4(0.3,0.6) | 0.3(0.2,0.5) | 0.061 |
| **HGB (g/L, IQR)** | 81.0(68.0,98.0) | 86.0(69.0,102.0) | 81.0(68.0,97.8) | 0.613 |
| **PLT (10^9^/L, IQR)** | 170.5(105.3,232.8) | 168.0(110.3,235.3) | 170.5(105.3,228.5) | 0.919 |
| **PCT (ng/ml, IQR)** | 0.1(0.1,0.3) | 0.1(0.1,0.2) | 0.1(0.1,0.3) | 0.941 |
| **ALB (g/L, SD)** | 31.5±5.5 | 31.9±5.3 | 31.5±5.6 | 0.762 |
| **ALT (U/L, IQR)** | 28.9(16.2,54.2) | 34.4(18.2,53.8) | 28.9(16.0,54.2) | 0.449 |
| **AST (U/L, IQR)** | 37.4(27.2,62.8) | 37.9(32.8,79.5) | 37.4(26.8,61.9) | 0.368 |
| IQR: interquartile range, SD: standard deviation, NRTIs: nucleoside reverse transcriptase inhibitors, INSTIs: integrase strand transfer inhibitors, NNRTIs: non-nucleoside reverse transcriptase inhibitors, CRP: C-reactive protein, WBC: white blood cell, MONO: monocytes, HGB: hemoglobin, PLT: platelets, PCT: procalcitonin, ALB: albumin, ALT: alanine aminotransferase, AST: aspartate aminotransferase. | | | | |
